# Supplementary material for: Spin-induced multiferroicity in the binary perovskite manganite Mn2O3
Source: Nat Commun. 2018 Jul 31;9:2996. doi: 10.1038/s41467-018-05296-0 (PMC6068161; doi:10.1038/s41467-018-05296-0)
Supplement: Supplementary file 1 — Supplementary Information [file 41467_2018_5296_MOESM1_ESM.pdf]

Supplementary Information

Spin-induced multiferroicity in the binary perovskite manganite Mn_2O_3

Cong et al.

Magnetic structure in the high-temperature phase

Atom	$M_i(\mathbf{k}_0)[\mu_B]$	$M_i(3\mathbf{k}_0)[\mu_B]$
Mn1	0.8(1)	
Mn2	2.85(3)	0.2(1)
Mn3	2.84(3)	0.2(1)
Mn4	1.6(1)	-2.1(3)

Supplementary Table 1 Fourier coefficients of the spin density waves localized on the four symmetry distinct Mn-sites of the parent *R-3* ($2\sqrt{2}a_p \times 2\sqrt{2}a_p \times \sqrt{3}a_p$) structure, refined from the data collected at T = 60 K. Magnetic moment on any atom within or outside the unit cell can be obtained by the relation: $M_i(\mathbf{k}_0)\cos(2\pi \cdot \mathbf{k}_0 \cdot (\mathbf{r}_i + \mathbf{t}))$ $+M_i(3\mathbf{k}_0)\cos(2\pi \cdot 3\mathbf{k}_0 \cdot (\mathbf{r}_i + \mathbf{t}))$, where $\mathbf{r}_i$ and $\mathbf{t}$ are the position of i-th atom in a parent cell and lattice translation, respectively, $\mathbf{k}_0$=(0,0,9/8) and $3\mathbf{k}_0$=(0,0,3/8). The $R_I\bar{3}$ magnetic symmetry forces all the magnetic atoms to have only cosign term. Coordinates of the Mn atoms in the parent structure: are as follows: Mn1 (0,0,0), Mn2(0.5,0,0), Mn3(0.5,0,0.5) and Mn4(0,0,0.5). Reliability factors for the WISH data set collected at the detector bank with average 2θ value of 58°: R_{Bragg}(nuclear)=4.15%, R_{Bragg}(magnetic)=6.93%.

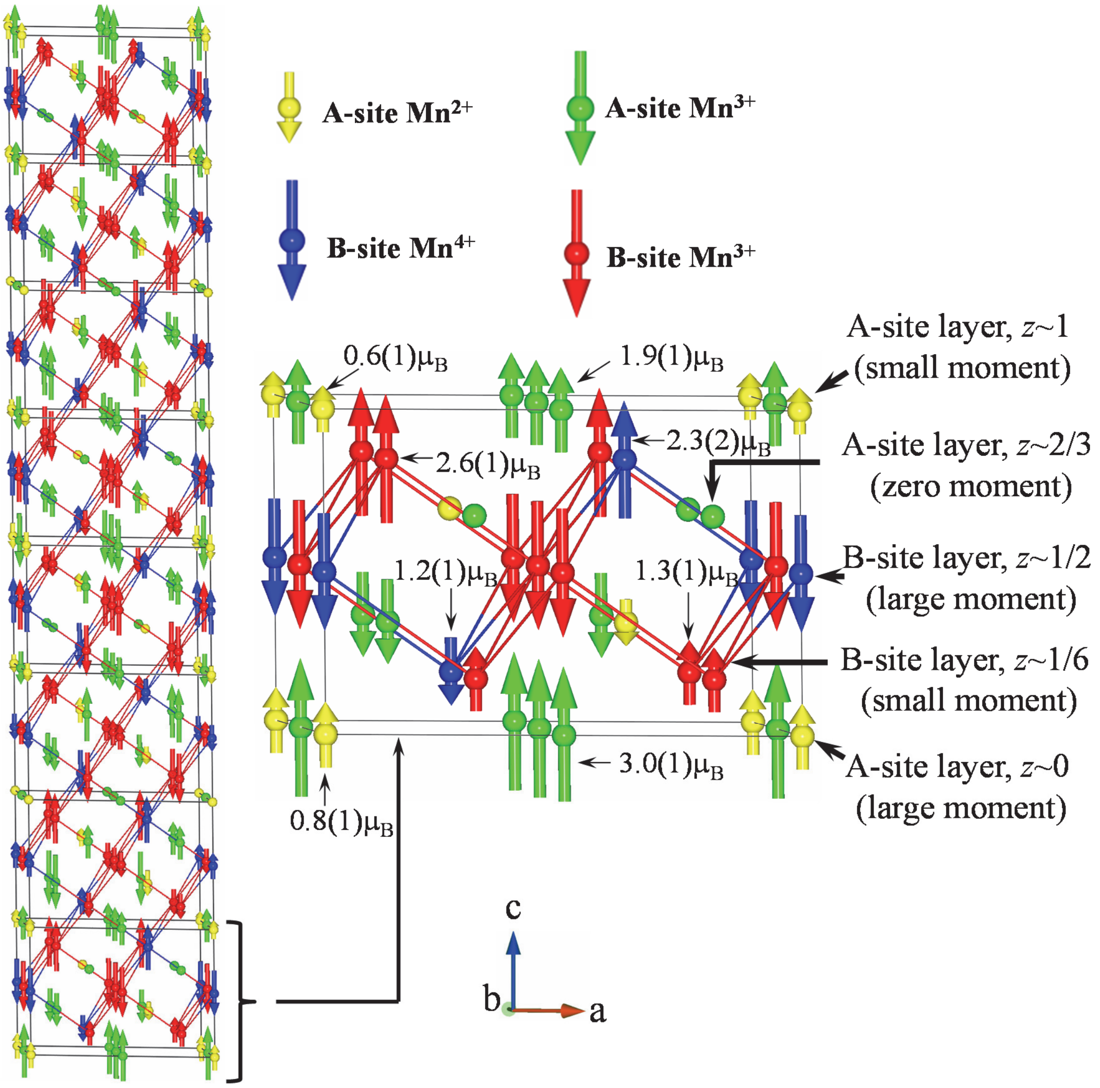

Supplementary Figure 1 Magnetic structure of the commensurate high-temperature phase (49 K < T < 101 K) of the binary perovskite Mn_2O_3. The structure is a longitudinal spin density wave with the unit cell eight times bigger than the cell of the parent *R-3* ($2\sqrt{2}a_p \times 2\sqrt{2}a_p \times \sqrt{3}a_p$) structure. It combines two types of the B-site and three types of the A-site Mn-layers stacked along the *c*-axis and having constant moments within the layers (the moment sizes are given for $T = 60$ K).

Magnetic structure in the low-temperature phase

Atom		$\mathbf{k}_0$	$\mathbf{k}_{1+}$	$\mathbf{k}_{1-}$	$\mathbf{k}_{2+}$	$\mathbf{k}_{2-}$	R_{tot}[μB]
Mn1	R_k=I_k[μB]	1.35(4)	-0.5(1)	-0.5(1)	0.09(4)	0.09(4)	1.53(8)
	φ_k	0	0.25	0.25	0.5	0.5	
Mn2	R_k=I_k[μB]	3.12(2)	1.00(3)	1.00(3)	0.16(1)	0.16(1)	3.44(3)
	φ_k	0	0.25	0.25	0.5	0.5	
Mn3	R_k=I_k[μB]	3.12(2)	1.00(3)	1.00(3)	0.16(1)	0.16(1)	3.44(3)
	φ_k	0.622	0.872	0.872	0.122	0.122	
Mn4	R_k=I_k[μB]	1.34(4)	1.5(1)	1.5(1)	0.8(1)	0.8(1)	3.0(2)
	φ_k	0.622	0.872	0.872	0.122	0.122	

Supplementary Table 2 Fourier coefficients of the phase modulated incommensurate ground state (T = 1.5 K), decomposed into a set of cycloidal/helical components: $\sum_{\mathbf{k}=\mathbf{k_0},\mathbf{k_{1\pm}},\mathbf{k_{2\pm}}}(R_k\,\hat{\mathbf{v}} + i\,I_k\,\hat{\mathbf{w}})e^{-2\pi i(kt+\varphi_k)} + (R_k\,\hat{\mathbf{v}} - i\,I_k\,\hat{\mathbf{w}})e^{2\pi i(kt+\varphi_k)}$, where R_k and I_k are real and imaginary parts of the Fourier coefficients, respectively, t is lattice translation and φ_k is magnetic phase. $\hat{\mathbf{w}}$ and $\hat{\mathbf{v}}$ are unit vectors, defining the spin plane. The first one was arbitrary chosen to be along the (100) direction (due to the metrically hexagonal cell, the refinement was not sensitive to the in-plane component), the second one was constrained to be in the plane perpendicular to this direction and was found to make an angle ~20(2)^o with the c-axis. To keep the moments constant upon a lattice translation, R_k and I_k were constrained to be equal (circular cycloid/helix). The four symmetry distinct Mn atoms in the parent structure follow the same notations as in the Supplementary Table 1. The propagation vectors: $\mathbf{k}_0$=(0,0,1.2439(3)), $\mathbf{k}_{1+}$=(0,0,1.9940(3)), $\mathbf{k}_{1-}$=(0,0,0.4940(3)), $\mathbf{k}_{2-}$=(0,0,-0.2560(3)), $\mathbf{k}_{2+}$=(0,0,2.7440(3)). Reliability factors for the WISH data set collected at the detector bank with average 2θ value of 58°: R_{Bragg}(nuclear) =4.67 %, R_{Bragg}(magnetic)=4.65%.

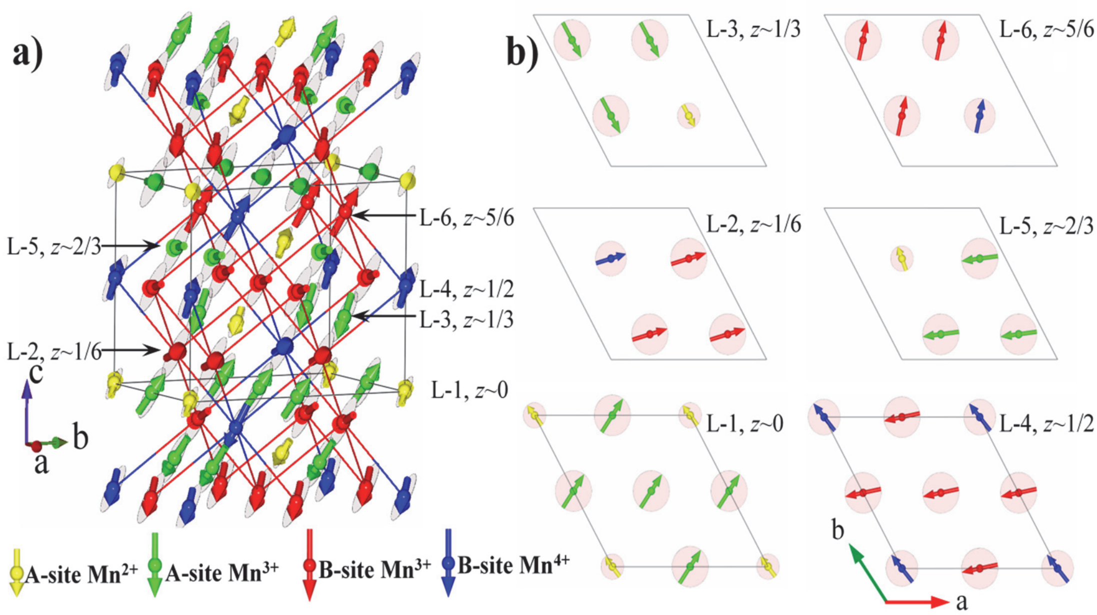

Supplementary Figure 2 (**a**) Magnetic structure of the incommensurate low-temperature phase (T < 49 K) of the binary perovskite Mn_2O_3. The structure combines both cycloidal and helical components, resulting in a general position of the plane containing spins (spin plane). (**b**) The magnetic structure presented as a set of layers (L-1, ... L-6) with different z-coordinates and the spin plane tilted to be with the (ab)-plane for clarity.

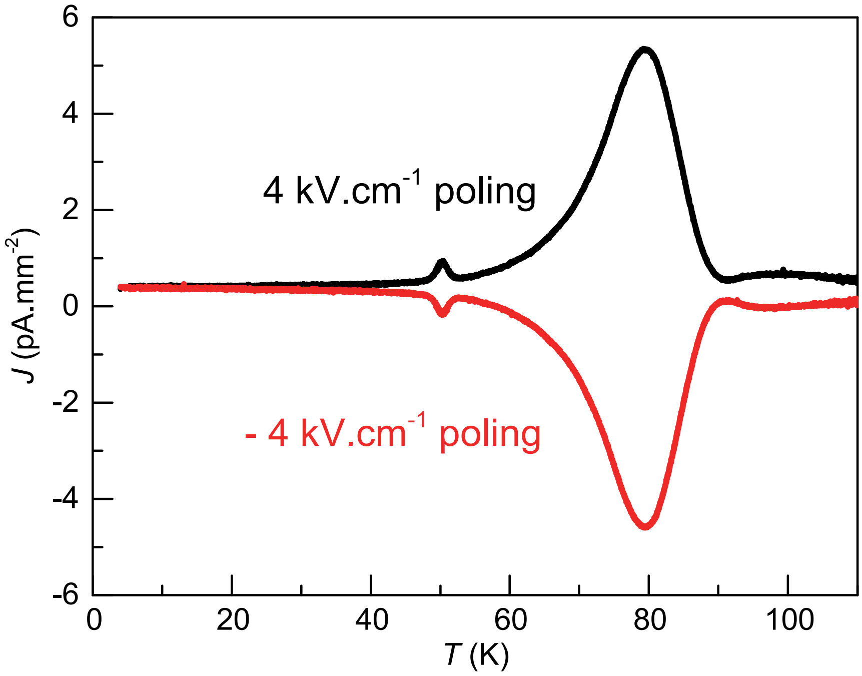

Supplementary Figure 3 The pyroelectric current data in the temperature range of 2 to 120 K with both positive and negative E-field poling. Two pyroelectric peaks are observed. The peak around 50 K is consistent with the magnetic transition at T_1. The pronounced peak around 80 K which does not coincide with any magnetic or structural phase transition is ascribed to extrinsic factors such as space trapped charges at grain boundaries.

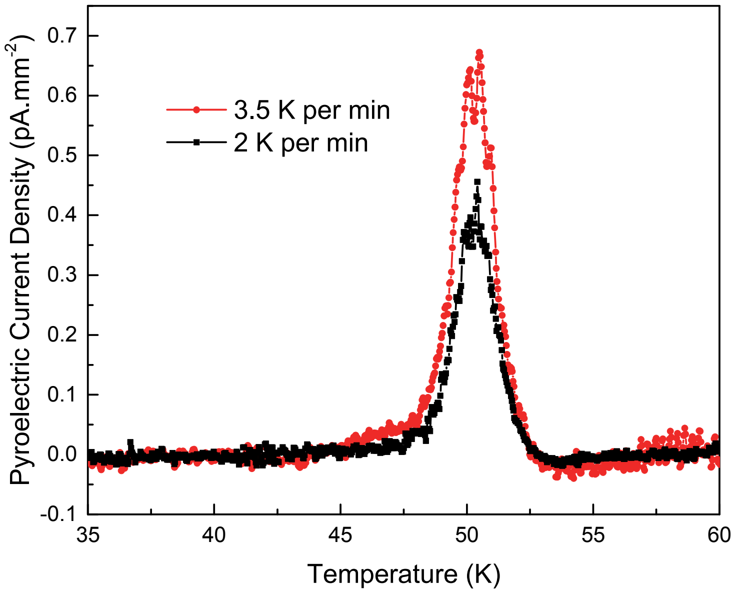

Supplementary Figure 4 Pyroelectric current as a function of temperature with different heating rate. The peak position does not shift with the heating rate, suggesting intrinsic ferroelectricity below ~ 50 K.

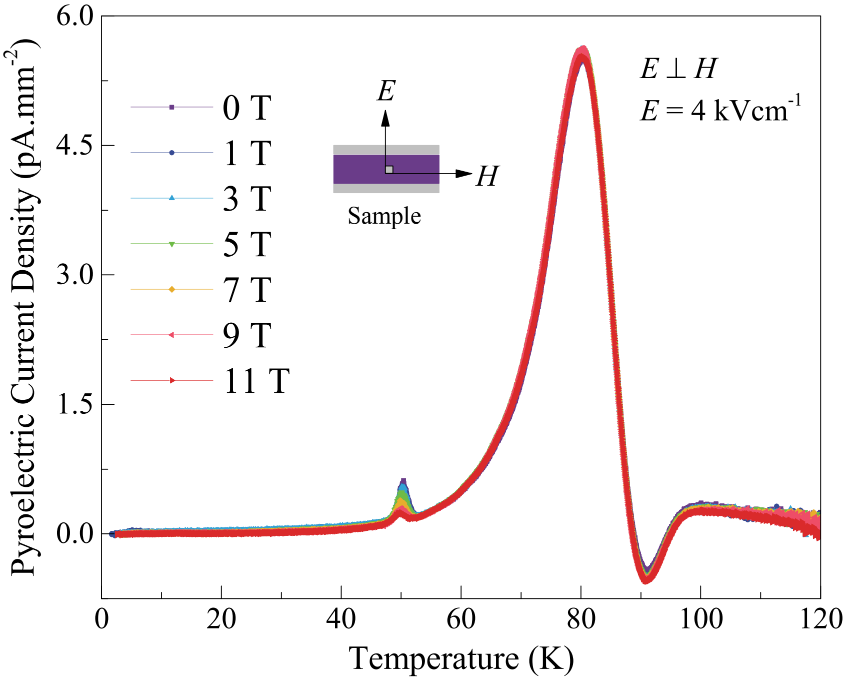

Supplementary Figure 5 Pyroelectric current as function of temperature from 2 to 120 K under different magnetic fields. The magnetic field is applied at 120 K in the presence of the poling *E*-field and is kept constant during the pyroelectric measurement. The peak at 50 K is suppressed by external magnetic fields but the broad peak around 80 K does not change with magnetic fields.

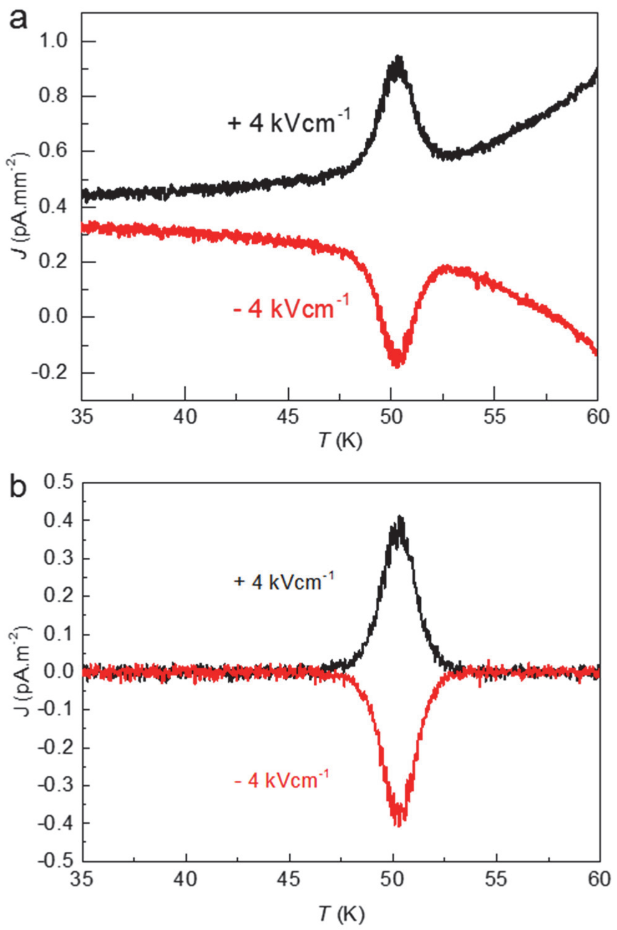

Supplementary Figure 6 Procedure of electric polarization calculation. (a) Raw data of the measured pyroelectric current. (b) Pyroelectric current after the background subtraction; the background is determined by a polynomial fitting to the base line. The electric polarization is calculated by integration of the pyroelectric current as function of time in (b).

Supplementary Note 1

Second harmonic generation measurements

The ferroelectricity in the binary perovskite Mn_2O_3 is further checked by polarized second harmonic generation (SHG) technique with a 90° reflection geometry as schematically shown in Supplementary Figure 7.

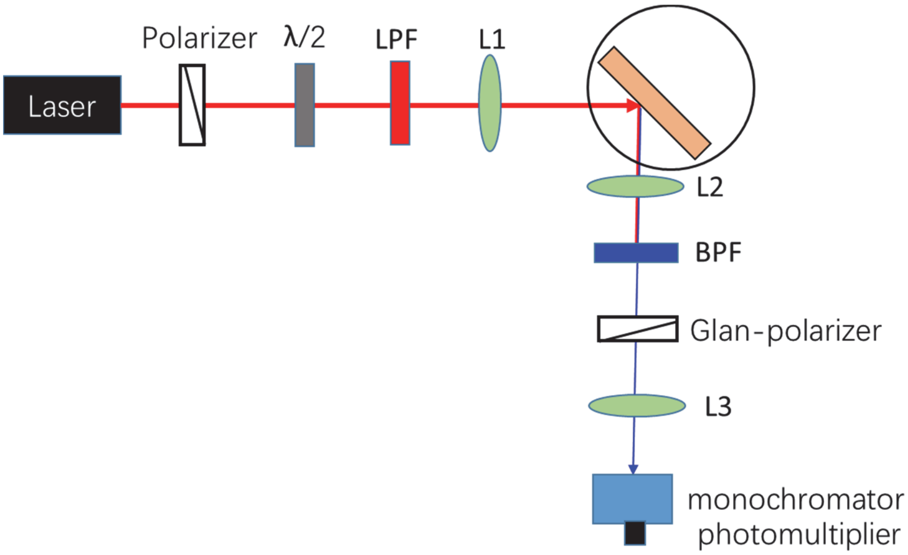

Supplementary Figure 7 A schematic view of the experimental coordination for the SHG measurements. The fundamental light is supplied by a Ti: sapphire oscillator with 80 MHz repetition rate and 100 fs bandwidth. The generated SH (~ 400 nm) from samples is filtered by color filters and a monochromator, and then detected by a photomultiplier tube (PMT). During the measurements, the polarization of both incident and output light beam can be manipulated respectively by polarizer components, such as Glan prism.

Supplementary Figure 8(a) and 8(b) present the SH polarization analysis diagram with *p*-polarization incidence (Pin) and *s*-polarization incidence (Sin), respectively. It is found that the SH signals obtained at high temperatures (120 and 70 K) are almost zero for both

Pin and Sin geometries. With temperature cooling down to 40 K, there exists obvious SH output from the binary perovskite Mn_2O_3 sample with two-fold symmetry, which indicates the emergence of electric polarization at low temperatures. The SHG results further confirm that the broad peak of pyroelectric current at high temperature (80 K) is due to extrinsic factors and the spin-induced ferroelectricty below T_1 = 49 K is real.

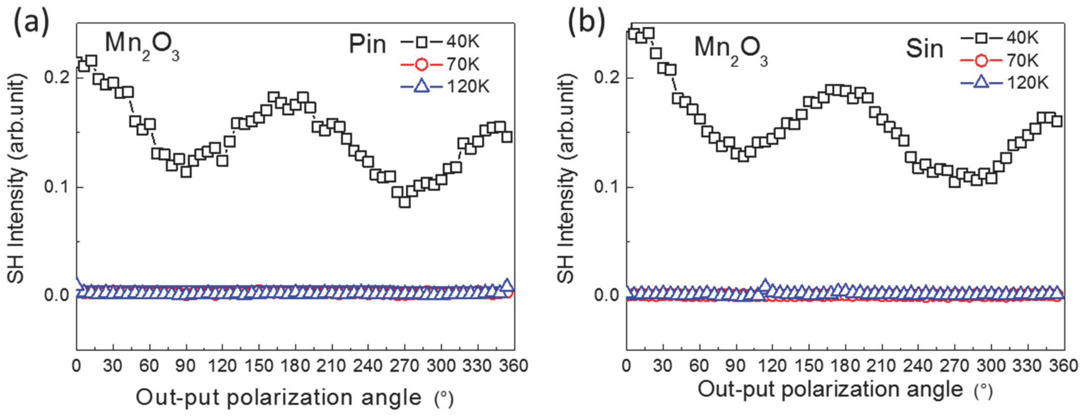

Supplementary Figure 8 Polarization angle dependence of SH signal with (a) Pin and (b) Sin geometries, respectively. The SHG is measured at different temperatures. The typical results obtained at 120, 70, and 40 K are presented in this figure.
